# Identification of swine influenza A virus and Stenotrophomonas maltophilia co-infection in Chinese pigs

**DOI:** 10.1186/1743-422X-9-169

**Published:** 2012-08-22

**Authors:** Dongjun Hou, Yuhai Bi, Honglei Sun, Jun Yang, Guanghua Fu, Yipeng Sun, Jinhua Liu, Juan Pu

**Affiliations:** 1 Key Laboratory of Animal Epidemiology and Zoonosis, Ministry of Agriculture, College of Veterinary Medicine, China Agricultural University, Beijing, 100193, People’s Republic of China; 2 Chinese Academy of Sciences Key Laboratory of Pathogenic Microbiology and Immunology (CASPMI), Institute of Microbiology, Chinese Academy of Sciences, Beijing, 100101, China; 3 Shandong Animal Disease Control Center, Jinan, 250022, Shandong Province, P.R. China; 4 College of Veterinary Medicine, China Agricultural University, No.2 Yuanmingyuan West Road, Beijing, 100193, China

**Keywords:** Swine influenza virus, *S. maltophilia*, Co-infections, Guinea pigs

## Abstract

**Background:**

Influenza virus virulence can be exacerbated by bacterial co-infections. Swine influenza virus (SIV) infection together with some bacteria is found to enhance pathogenicity.

**Methods:**

SIV-positive samples suspected of containing bacteria were used for bacterial isolation and identification. Antimicrobial susceptibility testing was performed by disc diffusion methods. To investigate the interaction of SIV and the bacteria in vitro, guinea pigs were used as mammalian hosts to determine the effect on viral susceptibility and transmissibility. Differences in viral titers between groups were compared using Student’s *t*-test.

**Results:**

During surveillance for SIV in China from 2006 to 2009, seven isolates (24.14%) of 29 influenza A viruses were co-isolated with *Stenotrophomonas maltophilia* from nasal and tracheal swab samples of pigs. Antimicrobial susceptibility testing showed that the bacteria possessed a high level of resistance towards clinically used antibiotics. To investigate the interaction between these two microorganisms in influencing viral susceptibility and transmission in humans, guinea pigs were used as an infection model. Animals were inoculated with SIV or *S. maltophilia* alone or co-infected with SIV and *S. maltophilia*. The results showed that although no transmission among guinea pigs was observed, virus–bacteria co-infections resulted in higher virus titers in nasal washes and trachea and a longer virus shedding period.

**Conclusions:**

This is the first report of influenza virus co-infection with *S. maltophilia* in the Chinese swine population. Increased replication of virus by co-infection with multidrug resistant bacteria might increase the infection rate of SIV in humans. The control of *S. maltophilia* in clinics will contribute to reducing the spread of SIV in pigs and humans.

## Background

Influenza A virus infection is a clinically and economically important pathogen causing respiratory disease in pigs worldwide. Swine influenza virus (SIV) subtypes H1N1, H3N2 and H1N2 can also cause zoonotic disease with flu-like symptoms in humans [1]. The recent emergence of the pandemic H1N1 virus was potentially of swine origin and provides a reminder that infection of pigs with influenza A viruses pose important public health concerns [2].

Influenza virus infections are usually exacerbated by secondary bacterial infections, and are one of the major causes of severe influenza pneumonia in humans, possibly due to the synergistic effect of these microorganisms during respiratory tract invasion [3,4]. It was reported that co-infection of SIV and bacteria such as *Streptococcus pneumoniae*, *Haemophilus influenzae* or *Staphylococcus aureus* leads to higher morbidity and mortality in mammals [5-7].

From our swine influenza surveillance work from 2006 to 2009, samples were inoculated into specific pathogen free (SPF) eggs for viral isolation [8]. It was found that inoculated embryonated eggs of some SIV-positive isolates died during the isolation procedure. Visual inspection of these eggs showed that the allantoic fluid was turbid. From previous experience, the majority of SIVs do not cause egg death, as any bacteria in the swab are killed by the multiple antibiotics in the transport medium. Thus, we hypothesized that the collected samples contained bacteria that was not killed by antibiotics in the transport medium, and that these multidrug resistant bacteria survived by escaping antibiotic treatment, and might contribute to an increased chance of co-infection with influenza virus due to the enhancement of viral susceptibility and interspecies transmission. In light of reports of SIV infections in humans, especially in individuals that have had direct contact with pigs [9], this study aimed to identify co-infecting bacteria and investigate their interactions with SIV in viral replication and transmission using a guinea pig infection model.

## Results

### Bacterial isolation and identification

SIV-positive samples still containing bacteria after treatment with antibiotics in viral transport medium were subjected to bacterial isolation and identification. All of the isolates produced typically similar small (<1 mm), circular, convex, colorless colonies. Analysis with the RiboPrinter Microbial Characterization System demonstrated that all these samples contained *Stenotrophomonas maltophilia*. The isolates displayed a typical API 20 NE profile (1-4-3-2-3-4-1) with a good identification score of 99.9% at the species level (Table 1). Furthermore, the similarity of 16S rRNA sequences from tested samples and *S. maltophilia* [GenBank: HQ246220] was ≥99%. Thus, these findings demonstrated that these isolates were *S. maltophilia*. Among the 29 isolates, seven (24.14%) viruses were co-infected with *S. maltophilia*: swine/Guangdong/7/06 (H3N2), swine/Shandong/133/07 (H3N2), swine/Fujian/43/07 (H3N2), swine/Guangdong/106/07 (H3N2), swine/Guangdong/211/2006 (H3N2), swine/Guangdong/109/2006 (H1N1) and swine/Guangdong/33/2006 (H1N1). Further antimicrobial susceptibility testing using the disc diffusion method showed that the bacteria were multidrug resistant and possessed a high degree of resistance towards clinically used antibiotics such as streptomycin, sulfadimidine, gentamicin, trimethoprim, ampicillin, amoxicillin, novobiocin, nitrofurantoin, cefotaxime, ceftazidime.

**Table 1:** Biochemical profile of the isolate with API 20 NE system

**Reactions/Enzymes**	**Results**	**Reactions/Enzymes**	**Results**
Reduction of nitrates	+^*^	Assimilation (mannitol)	−
Indole production (tryptophane)	−	Assimilation (N-acetyl-glucosamine)	+
Fermentation (glucose)	−	Assimilation (maltose)	+
Arginine dihydrolase	−	Assimilation (Potassium gluconate)	−
Urease	−	Assimilation (capric acid)	−
Hydrolysis (β-glucosidase) (esculin)	+	Assimilation (adipic acid)	−
Hydrolysis (protease) (gelatin)	+	Assimilation (malate)	+
β-galactosidase	+	Assimilation (trisodium citrate)	+
Assimilation (glucose)	−	Assimilation (phenylacetic acid)	−
Assimilation (arabinose)	−	Cytochrome oxidase	−
Assimilation (mannose)	+		

^*^+: positive result; −: negative result.

### Susceptibility in guinea pigs

Guinea pigs have recently been shown to be an alternative mammalian model for the study of human infection with influenza A virus, especially for the study of viral transmissibility [10]. Thus, we used guinea pigs to evaluate the susceptibility and transmissibility of SIV or SIV co-inoculated with *S. maltophilia*. Among the seven swine influenza isolates, A/Swine/Guangdong/7/06 (H3N2) belonged to a recent human-like lineage and possessed the closest genomic homology with human virus A/Moscow/10/99 [8]. This virus was therefore selected for the animal experiments.

**Figure 1:**
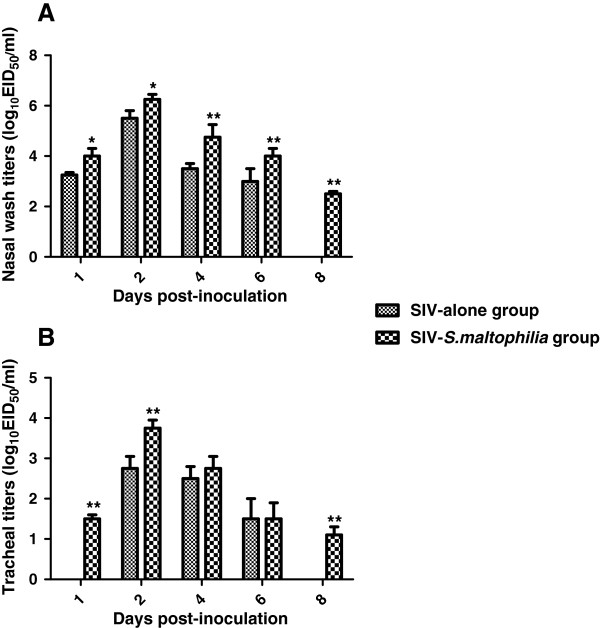
**Replication of influenza virus A/Swine/Guangdong/7/06 (H3N2) in guinea pigs.** Groups of 18 animals were inoculated intranasally with 100 μL (10^6^ EID_50_) of virus, or a 100 μL mixture of virus (10^6^ EID_50_) and *S. maltophilia* (1.5 × 10^7^ CFU). On days 1, 2, 4, 6 and 8 post-inoculation, three animals from each group were euthanized. Nasal wash, tracheas and lungs were collected for virus titration in eggs. **(A)** Virus titers in nasal washes. **(B)** Virus titers in tracheas. Each bar represents the virus titer expressed as mean log_10_ EID_50_/mL ± SD. The lower limit of detection was 10^1.5^ EID_50_/mL, or 10^1.5^ EID_50_/mL of tissue homogenate. Virus titers at each time point were compared between single virus-inoculated and virus-bacterial co-inoculated groups. * indicates statistically significant difference of means with p < 0.05, ** indicates statistically significant differences of means with p < 0.01.

During the 14-day experimental observation period, no weight loss was observed following infection with SIV or *S. maltophilia* either alone or as a co-infection. However, guinea pigs co-inoculated with the virus and bacteria showed depression, while no obvious disease signs were observed in the virus or bacteria-inoculated groups. *S. maltophilia* were recovered from the tracheas and lungs of the bacteria-inoculated groups but not from the DPBS control group, which suggested that *S. maltophilia* successfully infected the guinea pigs. Viruses were recovered from nasal washes and tracheas but not from lungs, with the highest viral titers occurring on day 2 post-inoculation (p.i.). The yields of infectious virus were higher in nasal washes than in tracheas. The viral replication kinetics in the nasal washes and tracheas are shown in Figure 1. The duration of virus replication was longer in the co-inoculated group and viruses could be detected on day 8 p.i. The virus titers in nasal washes in the co-inoculated group were significantly higher (P < 0.05) than in the group with single virus inoculation at all detection times. All guinea pigs inoculated with SIV showed seroconversion when tested on day 14 p.i. (Table 2).

**Table 2:** Virus, clinical signs, virus replication, transmission and seroconversion of guinea pigs

**Inoculum**	**Inoculated guinea pigs**			**Contact guinea pigs**		**Transmission**
	**Clinical signs**	Peak mean nasal wash titer ± SD (day)^**^	Seroconversion (HI titer range)^***^	Virus detected in nasal wash^****^	**Seroconversion (HI titer range)**	
Swine influenza virus (SIV)	None	5.50 ± 0.6 (2)	3/3^*^(40)	0/3	0/3	None
SIV + *S. maltophilia*	Depression	6.25 ± 0.4 (2)	3/3 (40–80)	0/3	0/3	None

^*^Number of positive guinea pigs/total number; ^**^Peak nasal wash titers are expressed as the mean log_10_EID_50_/mL ± SD; ^***^Serum was collected on day 14 p.i. for the virus inoculated group and on day 21 p.i. for the contact group, and homologous strains were used to detect SIV seroconversion with chicken red blood cells in hemagglutination inhibition assays. ^****^The lower limit of detection was 10^1.5^ EID_50_/mL.

### Transmissibility in guinea pigs

Virus was not detected in any of the contact animals and seroconversion tests were all negative for SIV, suggesting that transmissibility did not occur in this experiment (Table 2).

### Histopathology in guinea pigs

**Figure 2:**
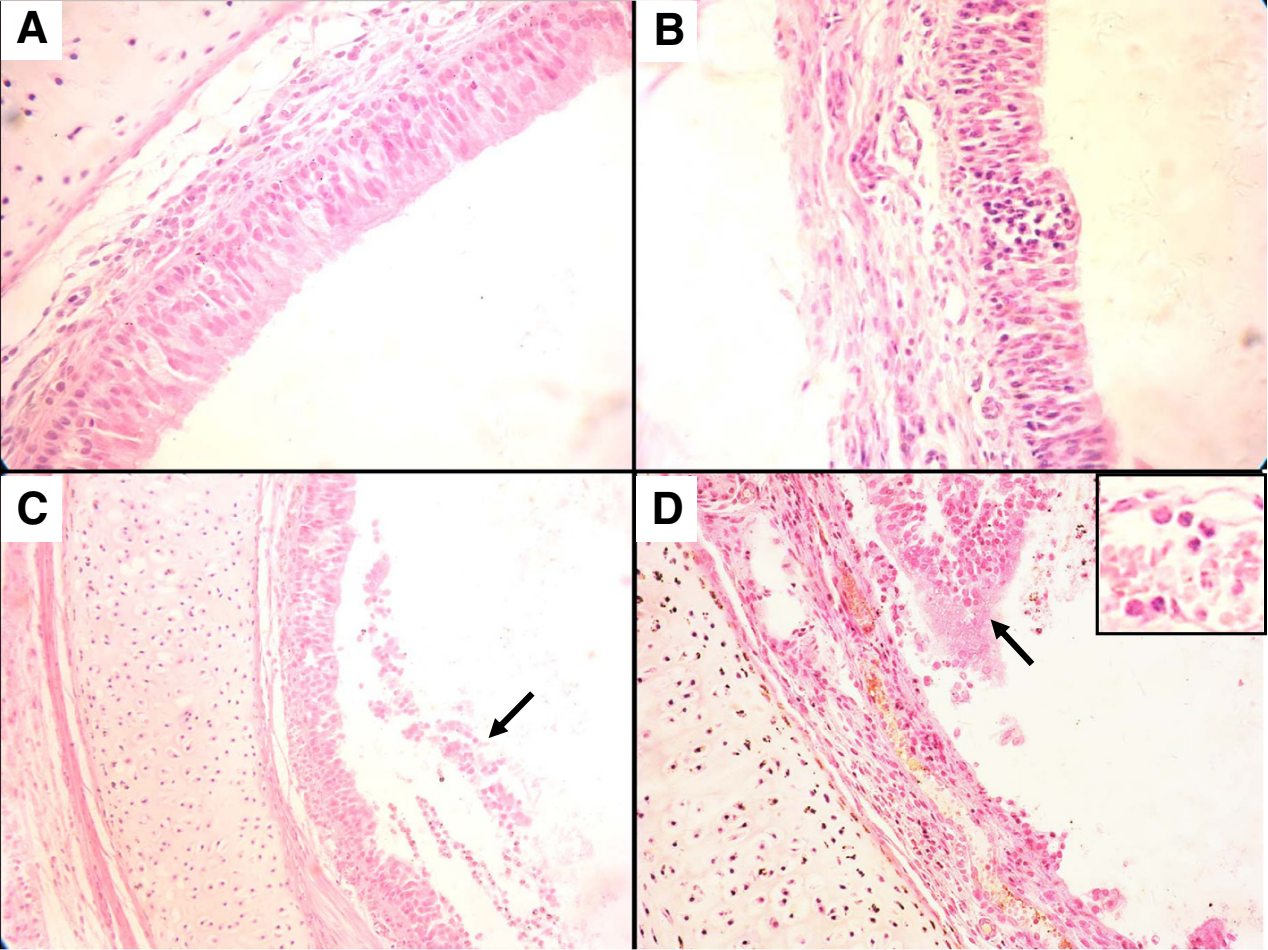
**Representative histopathological changes in HE-stained trachea from inoculated guinea pigs day 4 post-inoculation. (A)** Negative control. No apparent histopathologic lesions. **(B)** Bacteria-infected group. No apparent histopathologic lesions. **(C)** Virus-infected group. Dropout of mucous epithelium and inflammatory cell infiltration (solid arrow). **(D)** Co-infected group. Dropout of mucous epithelium cells, with many neutrophils and erythrocytes present (solid arrow). Tissue sections were observed under a microscope (Nikon, Japan). Magnification: (A, B, and D) × 400; C × 200.

To compare the histopathological changes in guinea pigs inoculated with SIV or *S. maltophilia* alone or by co-inoculation, the trachea and lungs were removed from infected animals on day 4 p.i., were fixed in 10% neutral buffered formalin and processed for routine histology. The representative histopathology is shown in Figure 2A-D. The histopathological changes in guinea pigs infected with virus and bacteria were more severe than in groups solely infected with virus or bacteria. Guinea pigs infected with both virus and bacteria showed severe pathological lesions in the trachea, characterized by severely damaged tunica mucosa tracheae. A number of mucous epithelial cell exfoliates, inflammatory cells, and erythrocytes were found in the trachea. Neutrophils were the predominant inflammatory cell when analyzed at high magnification (Figure 2D). The virus-infected groups showed mild pathological lesions in the trachea, which were characterized by dropout of mucous epithelium and few inflammatory cells adhering to the surface of the trachea (Figure 2C). There were no apparent histological changes observed in the trachea of the bacteria-infected and control groups (Figure 2A-B). In addition, no apparent histological changes were observed in the lungs of all the infected and control groups.

## Discussion

During the surveillance period (2006–2009), we found the first evidence for co-infection of SIV and *S. maltophilia* in pigs in several provinces in China, where the isolated *S. maltophilia* showed multidrug resistance. The co-infection of SIV and *S. maltophilia* has only previously been reported in Brazil [11].

*S. maltophilia* is an aerobic, non-fermentative, Gram-negative bacterium belonging to the genus *Stenotrophomonas*[12]*. S. maltophilia* is ubiquitous in aqueous environments, soil and plants, and frequently colonizes breathing tubes such as endotracheal or tracheostomy tubes, the respiratory tract and indwelling urinary catheters. It is a causal agent of infection and has gained considerable prominence in recent years as an important nosocomial pathogen associated with significant case/fatality ratios in debilitated or immunosuppressed patients [13-17].

In 2004, *S. maltophilia* was first reported in pigs from China [18], which was followed by several reports describing bacteria infecting both healthy and diseased pigs [19-21]. However, the colonization rate of swine with *S. maltophilia* in China was unknown, and co-infection of *S. maltophilia* and influenza A virus had not been previously reported in the Chinese swine population.

In this study, the synergistic effect of SIV and *S. maltophilia* caused depression in co-infected guinea pigs. Higher virus titers in nasal washes and tracheal samples and a longer period of virus shedding in nasal washes of co-infected animals was found in comparison with animals infected only with either virus or bacteria. In addition, the co-infected group showed more severe pathological lesions in the trachea than the other groups. This *in vivo* finding suggests that co-infection can enhance SIV replication during infection of guinea pigs. However, replication of the virus was still confined to the upper respiratory tract and was not found in the lungs, as is the case for most human influenza viruses [22-24]. Swine influenza viruses can cause disease in humans [1]. Several cases of co-infection of influenza A virus and *S. maltophilia* in humans have also been reported [11,25]. *S. maltophilia* is a multidrug resistant bacterium and possesses a high degree of resistance towards most commonly used broad-spectrum antibiotics, which is also confirmed in this study [26,27]. *S. maltophilia* can survive antibiotic treatment and co-infect hosts together with SIV, which can enhance viral replication and may increase the incidence of human infection with SIV, or SIV and *S. maltophilia*. During the treatment of SIV co-infection, controlling *S. maltophilia* will contribute to a reduction in illness and viral spread in pigs and human.

## Conclusions

Here, we present data providing the first evidence for co-infection of influenza A virus and *S. maltophilia* in Chinese pigs. We show that co-infection contributed to the virulence of SIV in experimental guinea pigs. SIV and *S. maltophilia* pose an important public health concern, we believe it is of interest to further investigate co-infections involving these two infectious agents.

## Material and methods

### Samples

From 2006 to 2009, 3,546 nasal and tracheal swab samples were collected for influenza surveillance in four main swine industrial provinces in China (Beijing, Fujian, Guangdong and Shandong). Twenty-nine strains of swine influenza A virus, including 19 H1N1, a single H1N2 and 9 H3N2 strains were obtained. Genetic characterization of these viruses was reported in a previous study [8]. Swab sample transport medium contained minimum essential medium (MEM), 2 × 10^7^ IU/L penicillin G, 1 × 10^7^ IU/L streptomycin, 100 mg/L gentamicin, 100 mg/L nystatin, 100 mg/L polymyxin B and 1000 mg/L sulfanilamide. SIV-positive samples suspected of containing bacteria (assessed by egg death and turbidity of the allantoic fluid) were used for bacterial isolation and identification.

### Bacterial isolation and identification

SIV-positive swab samples suspected of containing bacteria were streaked onto LB agar plates and incubated at 37°C for 24 h. Bacterial colonies were collected and identified using a RiboPrinter Microbial Characterization System (DuPont Qualicon, DE, USA) [28]. Biochemical characterization was performed using an API 20 NE (BioMerieux, France) and analyzed with Apiweb software (BioMerieux, France) according the manufacturer’s instructions [29]. To confirm the identified result, several colonies selected randomly were used as template for 16S rRNA sequencing as previously described [30]. Sequences were analyzed by Basic Local Alignment Search Tool (BLAST) for species identification as previously described [29].

### Antimicrobial susceptibility testing

Antimicrobial susceptibility testing was performed by disc diffusion methods recommended by the Clinical and Laboratory Standards Institute (CLSI). The antimicrobial discs tested included ofloxacin, levofloxacin, streptomycin, sulfadimidine, gentamicin, azithromycin, trimethoprim, ciprofloxacin, minocycline, ampicillin, amoxicillin, novobiocin, vancomycin, nitrofurantoin, cefotaxime, ceftazidime. (supplied by Tiantan Company of Pharmaceutical and Biological Products Development, Beijing, China). Determination of the antimicrobial susceptibility test was performed according to the manufacturer’s instructions, which followed the criteria of the CLSI. Reference strains *Escherichia coli* ATCC 25922 and *Pseudomonas aeruginosa* ATCC 27853 were used as quality control organisms in all antimicrobial susceptibility tests.

### Animals

To investigate the interaction of SIV and *S. maltophilia* in influencing viral susceptibility and transmissibility in mammalian hosts, SPF/VAF (virus antibody free) Hartley strain female guinea pigs weighing 300–350 g and serologically negative for influenza virus were used. Animal experiments were approved by the Beijing Association for Science and Technology, the approve ID is SYXK (Beijing) 2007–0023, and complied with the guidelines of Beijing laboratory animal welfare and ethical of Beijing Administration Committee of Laboratory Animals. Zoletil 100 (tiletamine-zolazepam; Virbac S.A., Garros, France) was used to anesthetize animals by intramuscular injection (10–15 mg/kg).

### Susceptibility in guinea pigs

Animals were inoculated with SIV or *S. maltophilia* alone or by co-infection of SIV and *S. maltophilia*. Groups of 18 animals were anesthetized and inoculated intranasally with 100 μL (10^6^ 50% egg infection dose, EID_50_) of virus, or 100 μL of 1.5 × 10^7^ colony forming units (CFU) *S. maltophilia*, or a 100 μL mixture of the virus (10^6^ EID_50_) and bacteria (1.5 × 10^7^ CFU). Animals inoculated with Dulbecco’s phosphate-buffered saline (DPBS) were used as controls. On days 1, 2, 4, 6 and 8 p.i., three animals from each group were euthanized. Nasal washes, tracheas and lungs were collected and a part of each organ was homogenized in DPBS-A (DPBS containing 2,000 U/mL penicillin G and 2.5 mg/mL streptomycin) and then 0.10 mg/mL vancomycin was added (the *S. maltophilia* isolate was sensitive to vancomycin) for virus titration by EID_50_ assay. Nasal washes were performed by instilling a total of 1 mL of DPBS-A into the nostrils and collecting liquid runoff into a sterile Petri dish. To confirm successful bacterial inoculation, tracheas and lungs were also collected for isolation and identification on day 4 p.i., as previously described [31]. Groups of the remaining three animals were observed for 2 weeks for body weight, signs of disease, and tested for SIV seroconversion on day 14 p.i.

### Transmissibility in guinea pigs

For the contact transmission study, groups of 15 naive guinea pigs were housed with the virus-inoculated, or virus and bacteria co-inoculated animals at 24 h p.i. and observed for 21 days. On days 2, 4, 6 and 8 p.i., the nasal washes, tracheas and lungs of three contact animals for each group were collected and titrated for EID_50_ assay. Groups of the remaining three animals were observed over 21 days for body weight and signs of disease, and tested for seroconversion on day 21 post-contact.

### Histopathology

On day 4 p.i., trachea and lung specimens from inoculated guinea pigs were fixed in 10% neutral buffered formalin, routinely processed, and embedded in paraffin. Sections (4-μm thickness) were stained with hematoxylin and eosin (HE).

### Statistical analysis

Differences in viral titers between single virus-inoculated and virus-bacterial co-inoculated groups were compared using Student’s *t*-test. A P-value less than 0.05 was considered statistically significant.

## Competing interests

The authors declare that they have no competing interests.

## Authors’ contributions

JP, DJH and YHB performed the research. JHL and JP developed the study plan and wrote the paper. JY collected samples and gathered clinical data. GHF and YPS analyzed the animal experimental data and edited the draft manuscript. HLS analyzed the histopathological data. All authors have read and approved the final manuscript.
